# Case report of bilateral testicular infarction due to severe bilateral epididymo-orchitis: A catastrophic complication causing castration

**DOI:** 10.1016/j.ijscr.2020.07.013

**Published:** 2020-07-15

**Authors:** William Ong Lay Keat, Sivaneswaran Lechmiannandan, Devindran Manoharan, Say Bob Lee, Premnath Nagalingam

**Affiliations:** a Department of Urology, Penang General Hospital, Jalan Residensi, 10990, Georgetown, Penang, Malaysia; b Deanery, RCSI & UCD Malaysia Campus, Jalan Sepoy Lines, 10450, George Town, Penang, Malaysia

**Keywords:** Epididymo-orchitis, Testicular infarction, Bilateral, Orchidectomy, Ischemia, Castration

## Abstract

**Introduction:**

Testicular infarction as a result of severe epididymo-orchitis is an uncommon urological emergency, with only a handful of reported cases in literature. We report an even rarer case of bilateral epididymo-orchitis complicated with testicular infarction, resulting in bilateral orchidectomy.

**Presentation of case:**

49 year old gentleman presented with fever, persistent, unresolving pain and scrotal swelling of two weeks duration. Despite close clinical monitoring, timely ultrasounds of the testis and antibiotics there was an inexorable progression to bilateral testicular ischemia.

**Discussion:**

This is only the second reported case of this nature in published literature. Epididymo-orchitis usually responds well to appropriate antibiotic therapy, although progression to testicular infarction is possible.

**Conclusion:**

Clinical presentation of persistent scrotal pain and oedema in cases of epididymo-orchitis should raise strong suspicion of testicular ischemia or infarction. Despite all efforts, progression to bilateral testicular infarction resulting in castration is a possible catastrophic outcome.

## Highlights

• A rare case of bilateral epididymo-orchitis complicated with bilateral testicular infarction.
• Presentation was fever with persistent, unresolved pain and scrotal swelling.
• Antibiotic therapy failed to halt disease progression of bilateral testicular ischemia.
• Bilateral orchidectomy performed as both testes were not viable, resulting in castration and lifelong testosterone replacement.

## 1 Introduction

An “acute scrotum” is a fairly common presentation for various scrotal pathologies, such as epididymitis, epididymo-orchitis, orchitis, testicular torsion, appendix of testis torsion and incarcerated inguinal hernia. Testicular infections are usually treated conservatively with oral or parenteral antibiotics [1]. Unlike testicular torsion, the incidence of testicular infarction secondary to epididymo-orchitis is low. Only a few case series and review articles of unilateral cases have been reported in the literature [2,3]. David Eisner and colleagues were the first to report a case of bilateral testicular infarction secondary to epididymitis [4].

We present this rare case of bilateral testicular infarction caused by severe non-resolving bilateral epididymo-orchitis, which eventually led to anorchia and lifelong testosterone replacement therapy. To our knowledge, this is only the second reported case of this nature in published literature. This work has been reported in line with the SCARE criteria [10].

## 2 Presentation of case

A 49-year-old gentleman, who was recently diagnosed with Hepatitis B and hypertension, presented with fever, scrotal pain and swelling of two weeks duration. He had consulted his private practitioner and was treated with a one-week course of oral ampicillin-sulbactam. As his symptoms persisted, he visited our emergency department for further management. He complained of persistent pain over his scrotum, which was associated with dysuria and cloudy urine. He had no hematuria or urethral discharge. On physical examination, he was not septic and was hemodynamically stable. The abdominal examination was unremarkable. The scrotum was erythematous and warm. Both epididymis and testes were noted to be swollen and tender on palpation. His prostate was normal in size and not tender on digital rectal examination. He recorded a temperature of 40 °C. Blood investigations showed mild leukocytosis (total white cells of 16.0 × 10^9^/l) and elevated C-reactive protein (CRP) of 257.1 mg/L, suggestive of a systemic infection. His hemoglobin and renal profile were normal. An urgent scrotal ultrasound was performed which revealed bilateral epididymo-orchitis with increased testicular and epididymal vascularity. He was admitted for commencement of analgesia and intravenous amoxicillin-clavulanic acid to treat the infection. Due to the persistent fever and no improvement in the scrotal swelling, the antibiotic was switched to intravenous fluoroquinolone (ciprofloxacin 400 mg twice a day). Clinical monitoring was continued.

**Fig. 1:**
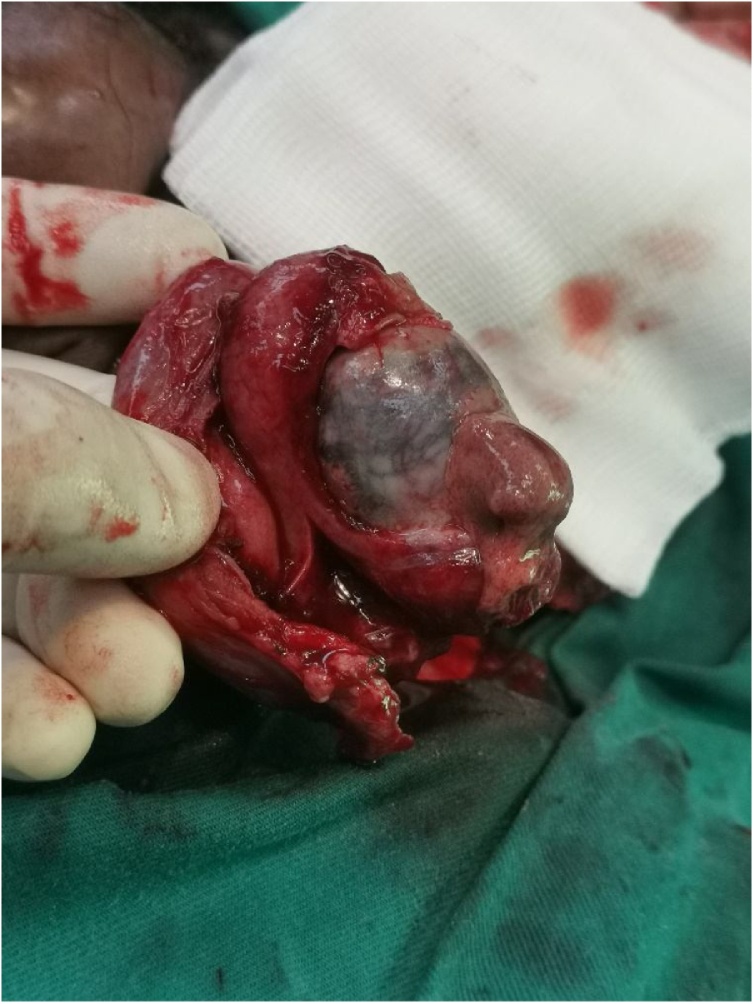
Image of the right non-viable testis with stab incision of tunica albuginea on the first surgery.

After five days of hospitalization, the patient’s scrotum remained swollen and his spermatic cord became progressively thickened and inflamed. Repeat ultrasound showed reduced blood flow to both testes suggestive of bilateral testicular ischemia. He underwent scrotal exploration. During surgery, the right testis appeared to be dusky and non-viable with thickened spermatic cord and no evidence of torsion (Fig. 1). A right orchidectomy was performed. Although the left testis appeared congested, it was spared with the hope of preserving it for testicular function. He was however counseled post operatively on the progressive risk of infarction requiring orchidectomy. Blood and pus culture revealed *Escherichia coli*, and his antibiotic was changed to intravenous cefuroxime based on the culture sensitivity results. The urine culture was negative. Daily dressings were performed for the scrotal wound. Throughout his subsequent hospitalization, he remained afebrile. His left scrotum, however, persisted to be tender and erythematous.

**Fig. 2:**
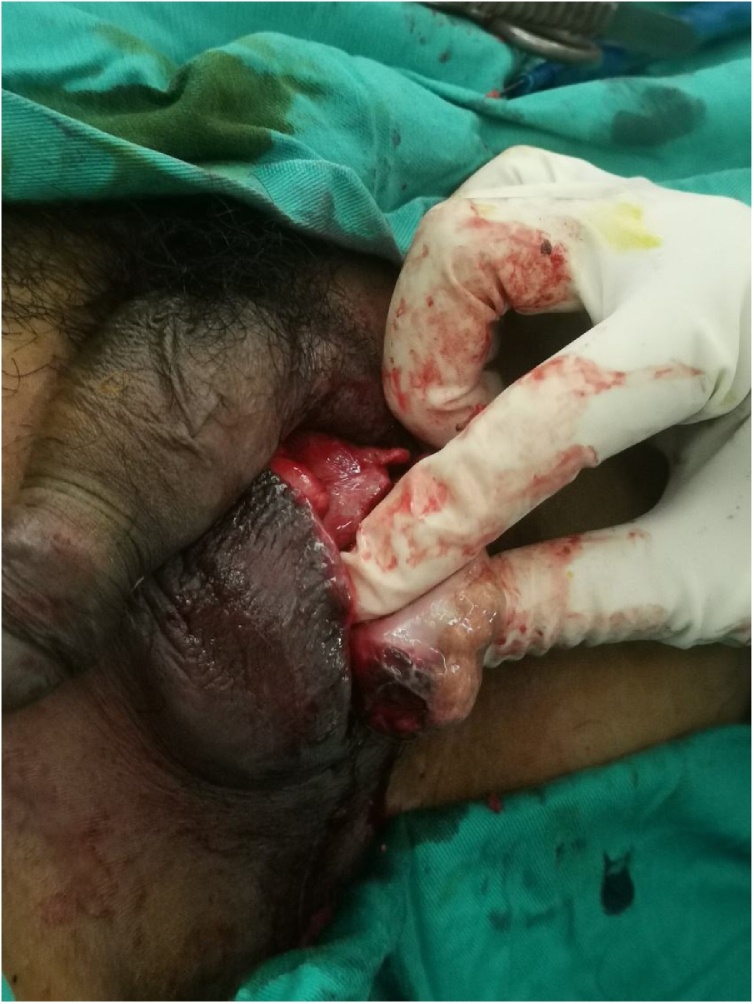
Image from the second surgery showing a suppurative non-viable left testis.

He underwent another scrotal exploration with informed consent for a possible left orchidectomy. The left testis was found to be suppurative and non-viable (Fig. 2). We proceeded with a left orchidectomy.

Histopathological report of both right and left testes confirmed testicular infarction. Microscopic examination revealed severe inflammation of the rete testis, ductuli efferentes and para-testicular soft tissues. The parenchyma of both testes was composed of closed packed non-viable seminiferous tubules with extensive necrosis. No acid-fast bacillus was detected in the specimen. He was subsequently discharged well and started on testosterone replacement (three monthly intramuscular testosterone injection) a week later. He was reviewed in our outpatient clinic a month later and was well with preserved erectile function. He was counseled for testicular prosthetic implants, but he was still indecisive.

## 3 Discussion

Epididymo-orchitis is a common inflammatory or infective condition, which usually responds well to appropriate antibiotic therapy, although there have been reported cases of the disease progressing to segmental or global testicular infarction. In a series of 610 cases reported by Mittemeyer et al., a testicular infarction rate of only 3–5% was seen [5]. On the other hand, Desai et al. reported severe testicular complications of frank infarction, suppurative necrosis and late atrophy in 39% of the 33 studied cases [6]. In 1991, Eisner and colleagues reported a case of bilateral testicular infarction following bilateral epididymitis [4]. To date, this appears to be the first and only reported case of bilateral involvement, with our case being the second, reflecting the rarity of this complication.

The exact pathogenesis of acute epididymo-orchitis causing testicular infarction still remains unclear. The testicular hypoperfusion theory postulates that the poor blood flow is due to compression of the testicular vascular pedicles by the swollen epididymis. Edema within the relatively non-compliant external spermatic fascia causes venous and lymphatic congestion in the testis and cord, promoting arterial occlusion and thrombosis [7]. Bacterial exotoxins and inflammatory infiltrates could play a pivotal role in the cumulative testicular edema and swelling [2].

While salvage rates for testicular torsions are high, based on clinical presentation and early recognition of signs, the same is not applicable in cases of epididymitis as the onset of testicular vascular compromise is unknown. Doppler ultrasonography of the testis, though not highly sensitive, is still the initial investigation of choice to determine presence of blood flow. Absence of intratesticular blood flow is a fairly reliable indicator of testicular ischemia. Another ultrasonic finding is reduced blood flow to the testis with increased peripheral flow to the scrotum. Other imaging modalities have been suggested to facilitate the diagnosis of testicular ischemia or infarction which include contrast enhanced ultrasonography (CEUS) and magnetic resonance imaging (MRI) [8]. MRI accurately assesses hypoperfusion for suspected testicular infarction but is not a practical investigative tool for routine use in our setting as it is not cost effective and not immediately available.

Despite close clinical monitoring and timely ultrasounds of the testis and antibiotic therapy, we were still unable to prevent this tragic outcome. Non-resolving testicular pain and swelling should raise awareness that testicular ischemia may have set in following an initial uncomplicated course of infection. Targeted antibiotics based on culture and sensitivity is still the mainstay of treatment in managing epididymo-orchitis, however this case serves a timely reminder that the rare complication of bilateral testicular infarct is still possible, resulting in catastrophic acquired anorchia from bilateral orchidectomy. Patients should be adequately counselled and be made aware of this. Tunica albuginea fasciotomy has been described as an operative measure to relieve testicular compartment syndrome, thus reducing vascular compromise [9]. Further evidence is awaited before this method can be recommended into mainline practice.

## 4 Conclusion

Clinical presentation of persistent scrotal pain and edema in cases of epididymo-orchitis should raise strong suspicion of testicular ischemia or infarction. Although doppler ultrasound is the standard imaging modality to assess testicular blood flow, its role in predicting early signs of ischemia is limited. Hospital admission and close clinical observation with broad spectrum antibiotic is still the mainstay treatment in managing severe acute infection of testes. Despite all these efforts, progression to bilateral testicular infarction resulting in castration is a possible catastrophic outcome.

## Declaration of Competing Interest

There are no conflicts of interest.

## Sources of funding

None to be stated.

## Ethical approval

Study is exempt from ethical approval by our institution.

## Consent

Written informed consent was obtained from the patient for publication of this case report and accompanying images. A copy of the written consent is available for review by the Editor-in-Chief of this journal on request.

## Author contribution

**William Ong Lay Keat**: Design, manuscript writing & literature review.

**Sivaneswaran Lechmiannandan**: Writing, literature review & editing.

**Devindran Manoharan**: Proof-reading & supervision.

**Lee Say Bob**: Proof-reading & supervision.

**Premnath Nagalingam**: Proof-reading & supervision.

## Registration of research studies

Not applicable.

## Guarantor

The corresponding author is the guarantor of submission.

## Provenance and peer review

Not commissioned, externally peer-reviewed.
